# MRI of paediatric liver tumours: How we review and report

**DOI:** 10.1186/s40644-016-0083-3

**Published:** 2016-08-15

**Authors:** Susan C. Shelmerdine, Derek J. Roebuck, Alexander J. Towbin, Kieran McHugh

**Affiliations:** 1 Department of Diagnostic Radiology, Great Ormond Street Hospital for Children NHS Foundation Trust, London, UK; 2 Department of Interventional Radiology, Great Ormond Street Hospital for Children NHS Foundation Trust, London, UK; 3 Department of Pediatric Radiology, Cincinnati Children’s Hospital Medical Center, Cincinnati, OH USA

**Keywords:** Liver, Hepatic, Mass, Lesion, Malignancy, Tumour, Paediatric, Hepatobiliary, MRI

## Abstract

Liver tumours are fortunately rare in children. Benign tumours such as haemangiomas and cystic mesenchymal hamartomas are typically seen in infancy, often before 6 months of age. After that age, malignant hepatic tumours increase in frequency. The differentiation of a malignant from benign lesion on imaging can often negate the need for biopsy. Ultrasound is currently the main screening tool for suspected liver pathology, and is ideally suited for evaluation of hepatic lesions in children due to their generally small size. With increasing research, public awareness and parental anxiety regarding radiation dosage from CT imaging, MRI is now unquestionably the modality of choice for further characterisation of hepatic mass lesions.

Nevertheless the cost, length of imaging time and perceived complexity of a paediatric liver MR study can be intimidating to the general radiologist and referring clinician. This article outlines standard MR sequences utilised, reasons for their utilisation, types of mixed hepatocyte specific/extracellular contrast agents employed and imaging features that aid the interpretation of paediatric liver lesions. The two commonest paediatric liver malignancies, namely hepatoblastoma and hepatocellular carcinoma are described. Differentiation of primary hepatic malignancies with metastatic disease and mimickers of malignancy such as focal nodular hyperplasia (FNH) and hepatic adenomas are also featured in this review..

Imaging should aim to clarify the presence of a lesion, the likelihood of malignancy and potential for complete surgical resection. Reviewing and reporting the studies should address these issues in a systematic fashion whilst also commenting upon background liver parenchymal appearances. Clinical information and adequate patient preparation prior to MR imaging studies help enhance the diagnostic yield.

## Background

Primary hepatic tumours account for only 1–2 % of all childhood cancers [1]. After 6 months of age a newly identified liver mass in a child will be malignant in the majority of cases [2]. The commonest primary malignant liver tumours in childhood include hepatoblastomas and hepatocellular carcinomas (HCC) with the remainder comprising comparatively rarer sarcomas (e.g. undifferentiated embryonal sarcomas, angiosarcomas and biliary rhabdomyosarcomas) [3]. Metastatic liver lesions in children, like in adults, are commoner than isolated primary hepatic lesions and commonly originate from neuroblastoma or Wilms’ tumours [4].

The aims of imaging are therefore directed at answering three main issues – confirming the presence of a liver lesion(s), defining it’s precise extent and whether it can be confidently characterised. The latter two findings will govern the need for biopsy or the approach for potential surgical management. Accurate radiological assessment is crucial at this stage, as it may negate the need for biopsy with benign processes [5], or alternatively direct further imaging such as a chest CT for evaluation of potential pulmonary metastases. The extent and number of malignant lesions also provides useful information in guiding the need for adjuvant chemotherapy and/or suitability for listing the patient for potential liver transplantation.

This article will describe the method by which to address the aforementioned factors whilst providing a general overview of the imaging findings in the two commonest malignant paediatric hepatic tumours and pitfalls in their diagnosis and follow-up imaging.

### Imaging approach

Ultrasound examination is the principal screening modality for identification of a suspected intra-abdominal mass in a child. Despite its ability to characterise the presence, size, solid component and vascularity of a liver mass - the mere presence of a lesion without an already established diagnosis should form the basis for further imaging with MRI. In some European centres, contrast enhanced ultrasound (CEUS) techniques may be adopted at this stage to help characterise likelihood of malignancy [6] and may inform the level of urgency for further MR assessment. CEUS is unfortunately not licenced for use in children and, although all the evidence suggests it is safe to use in young patients, it is not widely used as a consequence [7]. In addition, ultrasound is limited by its small field of view and subsequent difficulty in determining an accurate number or extent of liver lesions.

The advantages in utilising MRI for liver lesions include the lack of ionising radiation, good multi-planar spatial resolution (which in particular facilitates surgical planning) and excellent soft tissue characterisation. Nevertheless not all medical institutions possess the resources required to perform MRI studies in paediatric patients. This may be due to a variety of factors including cost, longer scanning times, the need for sedation in young patients, lack of local radiological expertise and machine availability. Where these limitations cannot be overcome or where urgent treatment and diagnosis are required, CT imaging may be performed, although the increased radiation burden and reduced soft tissue contrast makes it much less ideal [8]. If CT were performed to assess a hepatic mass lesion, we would advocate that a single portal venous phase CT is generally sufficient. In a child the size, vascularity and anatomical position of the lesion can all be assessed in the portal venous phase, and the other phases (non-contrast, arterial, delayed) add little diagnostic information [9].

When performing MR studies in children, adequate patient preparation can make a significant difference to the quality of the resultant images. Ideally patients should have nothing by mouth for 4 h prior to the study. Sedation or general anaesthesia may be needed (usually for those of ages less than 6–7 years) if the child is unable to hold his or her breath for longer than 20 s or if he or she cannot remain still for the approximately 45 min scan [10]. Occasionally, play specialists (Child Life) can be utilised in co-operative children as young as 5 years old to prevent sedation or general anaesthesia. Coils that are used can vary according to the size of the patient, but the smallest possible coil to achieve adequate coverage is recommended, and 8–32 phased-array surface channel coils are currently standard [11].

Despite the paucity of studies comparing diagnostic yield in liver lesion detection when utilising a 1.5 T versus 3 T magnet, our experience and those of other institutions are that a higher magnet strength produces better spatial resolution and is preferable in younger children when a choice exists [12, 13].

The length of the study can be variable and will be in part determined by patient co-operation (if un-sedated) and type of intravenous contrast agent used. Contrast agents for paediatric liver imaging fall predominantly into two subclasses, namely the typical extracellular agents (ECAs) used in most abdominal imaging, and mixed hepatocyte specific/extracellular agents. Both contrast agent subclasses include gadolinium-based media, containing a central gadolinium ion bound to a specific ligand, which determines the properties and anatomical distribution of the agent.

Mixed hepatocyte specific/extracellular agents are actively transported into hepatocytes and partially excreted through the biliary system. These therefore permit a more delayed ‘hepatobiliary phase’ imaging post administration allowing visualisation of the central biliary anatomy, thereby prolonging the length of the examination. Although routinely utilised with an excellent safety profile [14], they are technically ‘off label’ for use in paediatric liver imaging.

Examples of the two commonly used agents in this subclass include gadoxetate disodium (Gd-EOB-DTPA, marketed as Eovist/Primovist; Bayer HealthCare, Leverkusen, Germany) and gadobenate dimeglumine (Gd-BOPTA, marketed as Multihance; Bracco Imaging, Milan, Italy). Approximately 50 % of gadoxetate and 3–5 % of gadobenate is excreted through the biliary system, with the remainder excreted via the kidneys. Hepatobiliary phase imaging can be performed 20 min after injection of gadoxetate and 40 min after injection of gadobenate [15]. Both agents have been shown to help improve diagnostic confidence in identification of liver lesions in children, and in differentiating them from focal nodular hyperplasia (FNH) in particular [13, 16–18]. At our collective institutions, mixed hepatocyte specific/extracellular agents are routinely used during MR examinations in all patients with known or suspected hepatic lesion(s).

Standard liver imaging protocols usually consist of axial T1, axial (+/− coronal) T2 weighted fast/turbo spin echo sequences, axial 3D gradient recalled echo (GRE) sequences (out/in phase) or T1 DIXON GRE (to provide out/in phase with fat suppressed and water supressed images), axial (+/− coronal) balanced steady state free precession (SSFP), axial diffusion weighted images (DWI, b values 0, 100 and 800 s/mm^2^) and dynamic post contrast 3D or 4D GRE sequences (obtained in early arterial (10s after injection), arterial (at 20–30s after injection), portovenous (40–60s) and equilibrium (5 min)) and a delayed hepatobiliary phase [11, 19]. In order to save time, the pre-contrast T1 weighted images can be acquired as the first sequence, with the remaining sequences performed after contrast injection, and the delayed post-contrast hepatobiliary phase T1 weighted imaging performed last [18]. This capacity of multiple varied phases of contrast enhancement, with no additional radiation burden, is a significant reason why MRI is preferred to CT when evaluating liver lesions in children.

An informative article by Meyers et al. [11] details the paediatric liver MR sequences acquired at Cincinnati Children’s Hospital using gadoxetate disodium. The imaging protocol used at The Hospital for Sick Children, Toronto, where gadobenate dimeglumine is administered, can be found in the article by Chavhan et al. [18].

### The clinical request form

Prior to reviewing the imaging, crucial information gleaned from the clinical request form can already guide the radiologist towards a list of potential differential diagnoses [4]. Age plays a key factor as hepatoblastoma, hepatic hemangiomas, mesenchymal hamartomas and metastatic disease from neuroblastoma or Wilms’ tumours present mostly within the first 3 years of life [20] while hepatocellular carcinoma (HCC), FNH and hepatic adenomas occur chiefly in older children and adolescents.

Clinical tumour markers, if available to the radiologist, are also important. The alpha-fetoprotein (AFP) level in particular is key, as this is elevated in the majority of patients with hepatoblastomas and HCCs [21, 22]. It is noteworthy here too that it has recently become apparent that some ‘non-AFP secreting hepatoblastomas’, previously deemed higher risk disease, are actually hepatic rhabdoid tumours [23]. AFP has also been demonstrated to be an excellent marker in predicting tumour recurrence during follow-up with one 10 year retrospective study reporting no imaging identifiable relapses from hepatoblastoma without an abnormal elevation of serum AFP levels [24].

Patients with certain background medical histories (such as Beckwith Wiedemann syndrome, Familial adenomatous polyposis (FAP)) are prone to develop hepatoblastomas, whereas those with underlying glycogen storage diseases, biliary atresia, alpha-1 anti-trypsin deficiency and tyrosinaemia are prone to developing HCC. Children with a history of a treated solid tumour with chemotherapy and/or radiation therapy have also been shown to have a predisposition towards the development of FNHs [25].

Finally, it is also important to consider whether the patient is suffering from background liver disease, such as decompensated liver cirrhosis, which will reduce the level of hepatocyte uptake and biliary excretion of mixed hepatocyte specific/extracellular contrast agents [26] and hinder image interpretation. Unfortunately the degree to which this occurs does not appear to correlate to serum markers of liver function [27], so cannot be accurately predicted.

### Review of imaging/Useful sequences

After reviewing the clinical information, a systematic approach should be adopted during image review and reporting, ensuring that the following issues are addressed:

1. Lesion presence, number, anatomical location and imaging characteristics,
2. Background liver parenchymal appearances,
3. Evidence of metastatic spread, vascular or biliary complications,
4. Other non- hepatobiliary findings (such as presence of primary suprarenal or renal mass and/or lymphadenopathy).

**Fig. 1:**
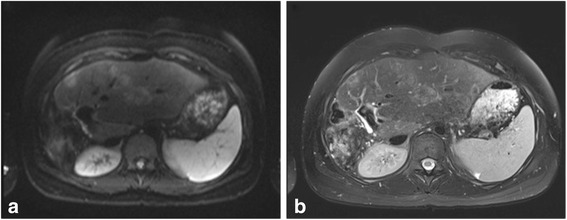
Multiple areas of focal nodular hyperplasia in a 17 year old patient with prior history of right hepatectomy for hepatoblastoma. (**a**) Diffusion-weighted imaging (b = 50 s/mm^2^) allows for improved detection of the multiple liver lesions than (**b**) the axial T2-weighted fat-saturated sequence

In identification of liver lesions, many authors have purported the usefulness of the low b-value diffusion weighted images (b = 50–100 s/mm^2^), with lesions being more apparent on this sequence than on the usual T2 weighted sequences [28–30] (Fig. 1).

The most crucial sequences in lesion differentiation include the pattern of contrast enhancement during dynamic and delayed hepatobiliary phases of imaging [11, 31]. Some typical and atypical findings of the commonest paediatric liver lesions are described later.

In adult patients, the use of ADC values in the characterisation of liver lesions has been limited [32]. There is a paucity of paediatric studies on this topic and therefore care should be taken when relying on diffusion-weighted characteristics for purposes of differentiating benign from malignant lesions [33].

Interpretation of the background liver parenchyma for diffuse liver disease, steatosis, fibrosis and iron deposition should also not be forgotten. Newer techniques such as MR elastography can help to quantify the degree of fibrosis, if needed, albeit little used in children to date [34, 35]. Multi-echo gradient imaging is helpful in assessing features such as steatosis and iron deposition (siderosis), with hepatic siderosis appearing more pronounced in image sequences obtained at longer TE times, as demonstrated by loss of signal intensity within the liver parenchyma [36, 37].

### Paediatric liver tumour characteristics

#### Hepatoblastoma

**Fig. 2:**
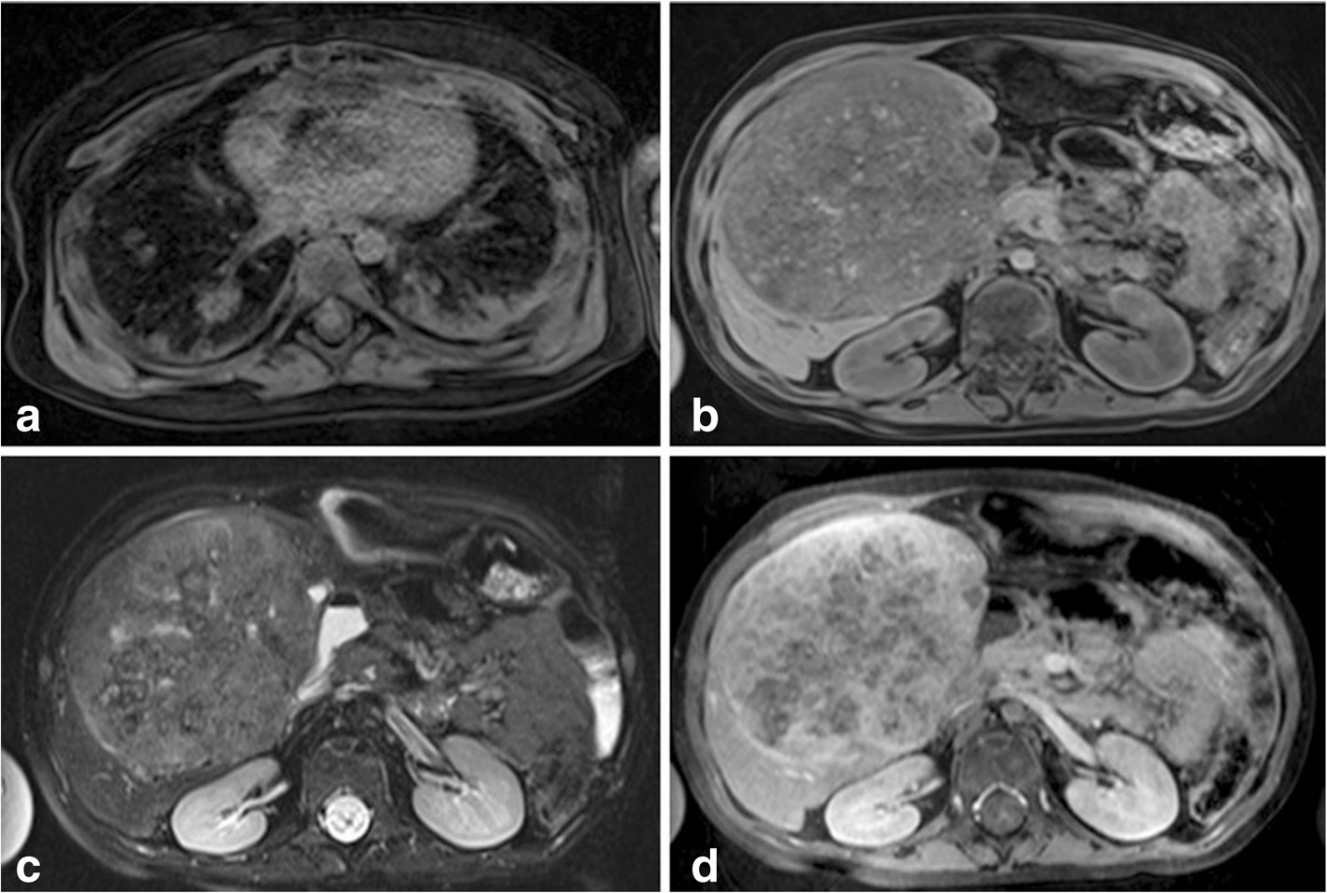
Hepatoblastoma in a 4 year old patient. The (**a**) axial T1-weighted fat saturated imaging of the lung bases demonstrates multiple pulmonary metastases. On (**b**) T1-weighted fat-saturated pre-contrast imaging, the hepatic mass has internal heterogenous signal intensity, with overall hypointensity compared to background liver parenchyma. It is mildly hyperintense compared to the liver parenchyma on (**c**) the T2-weighted fat saturated sequence. On (**d**) portal venous phase imaging, post gadobenate dimeglumine administration, the lesion has internal heterogeneous enhancement

A hepatoblastoma is the most common primary hepatic malignancy in the paediatric population. A typical hepatoblastoma on MRI is heterogeneously hyperintense on T2 weighted images, hypointense on T1 weighted imaging and enhances in a heterogenous fashion, although remaining on the whole hypointense when compared to the background liver parenchyma in all phases of enhancement [11, 38] (Fig. 2). Calcification within the mass is present in 50 % of cases and haemorrhage and necrosis may also occur in variable amounts leading to the heterogenous signal intensity [4].

Hepatoblastomas may also demonstrate atypical radiographic and clinical features [11]. Small cell undifferentiated subtypes of hepatoblastomas may not be associated with raised AFP levels [39]. Meyers et al. [11] report two cases of hepatoblastomas with avid enhancement during the hepatobiliary phase of imaging which, on pathology were reported to display teratoid features. The authors propose this enhancement may possibly relate to internal functioning hepatocytes. We have also noted in a few hepatoblastoma cases with fetal histology that the tumours have accumulated the hepatocyte specific agent, perhaps because these tumours contain some persisting hepatocyte functioning also.

Malignant paediatric hepatic tumours (predominantly hepatoblastoma, but also HCC) are staged prior to commencement of therapy according to the PRETreatment EXTent of tumour (PRETEXT) system, designed by the International Childhood Liver Tumor Strategy Group (SIOPEL) [40]. The staging has been shown to correlate closely to prognosis and survival (for children with hepatoblastoma and fibrolamellar hepatocellular carcinoma [41]) and has a good inter-observer reproducibility [42].

The latest version of this staging system, established in 2005, requires the radiologist to delineate the number of anatomical sections that are involved and those that are free of tumour. There are 4 anatomical sections described in the staging system which are divided based on groupings of the Couinaud’s segmentation of the liver. The PRETEXT score represents the number of contiguous sections that must be resected to completely excise the tumour.

**Fig. 3:**
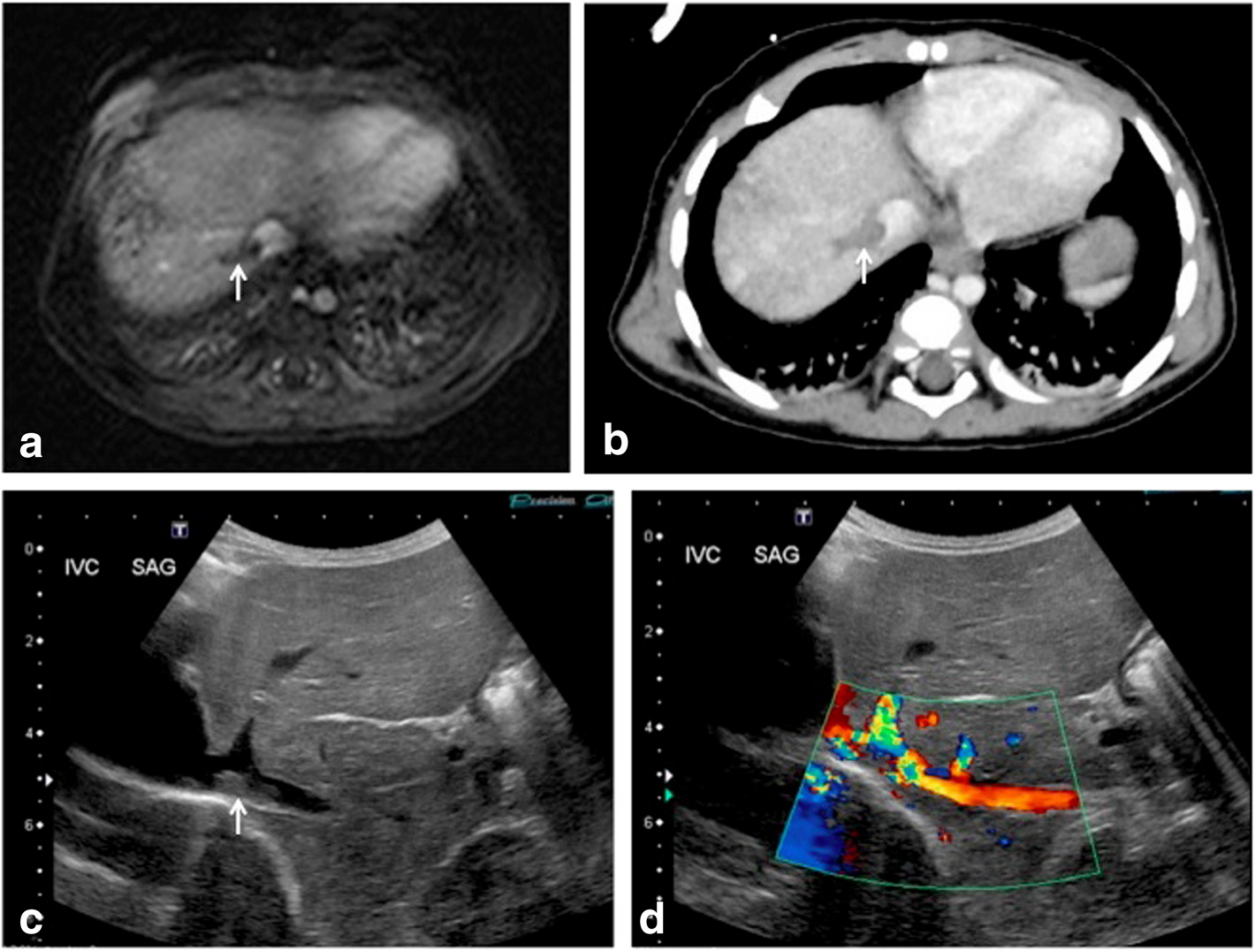
Metastatic hepatoblastoma with inferior vena cava (IVC) and right hepatic vein thrombus (*white arrows*) in a 2 year old boy. After administration of gadobenate dimeglumine, the (**a**) arterial phase T1-weighted fat-saturated imaging demonstrates a filling defect in the affected vessels. Thrombosis was subsequently confirmed and identified on (**b**) portal venous phase post-contrast imaging on CT and (**c**), (**d**) ultrasound imaging of the IVC with and without colour doppler overlay respectively

Additional imaging information for PRETEXT staging including hepatic, portal venous and IVC involvement (Fig. 3), lymph node enlargement, tumour rupture, presence of distant metastases and, albeit rare, extrahepatic abdominal spread of disease should be commented upon. If pre-operative chemotherapy has been instigated, it is recommended that repeat imaging be performed prior to any surgical intervention, and restaged in the same manner, recorded as the ‘POSTEXT’ staging [40].

#### Hepatocellular Carcinoma (HCC)

HCC is the most common primary hepatic malignancy in adolescence and the second most common primary paediatric malignancy of the liver. Paediatric HCC differs from adult-type HCC in several important ways. First, pre-existing liver disease is only present in 30–50 % of paediatric patients [38, 43]. While cirrhosis is the most common risk factor for developing HCC in adults, it is much less common in children living in the Western world, occurring in only 20–25 % of patients [38]. In addition, there are molecular differences of paediatric HCC including a higher rate of c-met gene mutations, a higher rate of loss of heterozygosity on chromosome 13q, and lower levels of cyclin D1 [38].

Unfortunately, the prognosis for HCCs occurring in children tends to be poorer than those observed in adults [4], with adolescent patients more commonly affected than young children [44]. Typical MRI features of HCC include avid arterial phase enhancement with wash-out on the portal venous phase of enhancement, compared to the background liver parenchyma. With hepatobiliary phase imaging, the lesion remains hypointense to adjacent liver, although rarely, atypical or early HCCs may demonstrate enhancement [27].

**Fig. 4:**
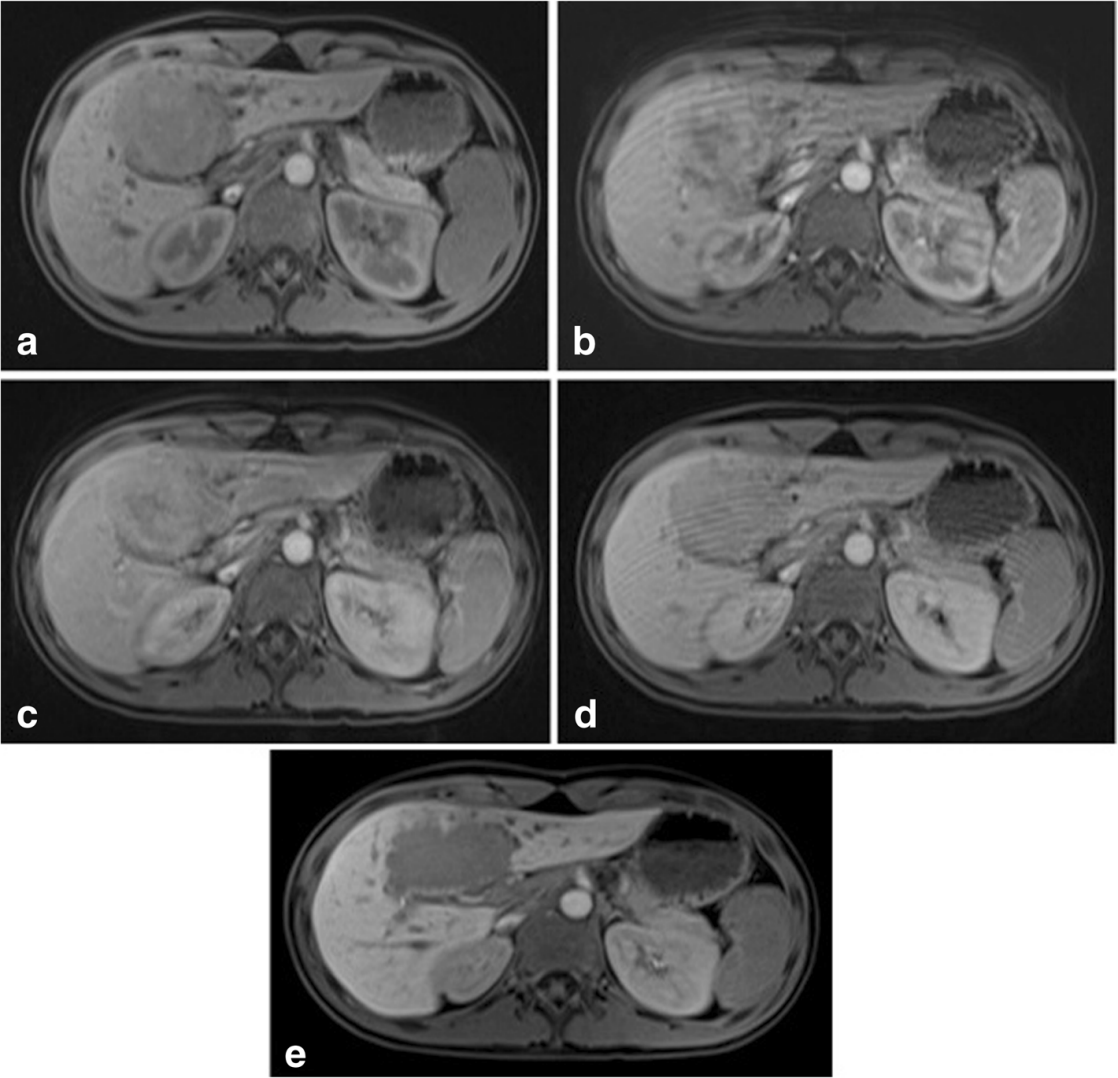
Fibrolamellar variant of hepatocellular carcinoma in a 15 year old patient. The hepatic lesion is hypointense to background liver parenchyma on (**a**) pre-contrast T1-weighted sequences with heterogenous internal enhancement after administration of gadoxetic acid in (**b**) arterial phase and (**c**) porto-venous phase imaging. There is gradual washout of contrast on the (**d**) 5 min delayed and (**e**) 20 min delayed imaging. Note the excretion of contrast material within the common bile duct allowing localisation of the mass and its relation to the biliary system, aiding surgical planning

The fibrolamellar variant of HCC is more commonly seen in young adults (Fig. 4). It is hyperintense on T2 weighted sequences and hypointense on T1 weighted sequences compared to background liver, with some demonstrating a hypointense central scar [20]. Post contrast, these tumours tend to demonstrate arterial enhancement with washout in the portovenous phase and remain hypointense in the hepatobiliary phase. There is not normally any enhancement of the central scar [26].

Fibrolamellar HCC is often included in a differential diagnosis list with focal nodular hyperplasia due to the presence of a central stellate scar. However, it can be differentiated from FNH based on the appearance of the central scar and the appearance of the tumour on the hepatobiliary phase. Fibrolamellar HCC has a hypointense scar on T2-weighted images while FNH has a hyperintense scar on the same sequence. On the hepatobiliary phase of enhancement fibrolamellar HCC is hypointense to the background liver while FNH is isointense to hyperintense to the background liver.

### Metastatic disease

Metastases from non-hepatic primary malignancies are commoner than those from primary hepatic tumours in general (such as those from Wilms’ tumours and neuroblastomas [44]). Liver metastases are typically hyperintense on T2 weighted images (although they can also be of intermediate signal intensity) and hypointense on noncontrast T1 weighted images [20]. Neither hyper nor hypovascular metastases enhance in the hepatobiliary phase of contrast [11].

Neuroblastoma metastases tend to demonstrate peripheral enhancement on arterial phase imaging with central progression of enhancement and peripheral washout on portal venous phase sequences. In some cases, they may also remain isointense [45]. A potential pitfall may arise when trying to differentiate such features from multifocal infantile hemangioendotheliomas [46], however the presence of additional metastases (such as bone lesions) or the primary tumour (which may be an adrenal, retroperitoneal or paravertebral mass) with MIBG avidity and elevated levels of urinary catecholamines will help to clinch the diagnosis.

### Focal Nodular Hyperplasia (FNH) and hepatocellular adenomas

**Fig. 5:**
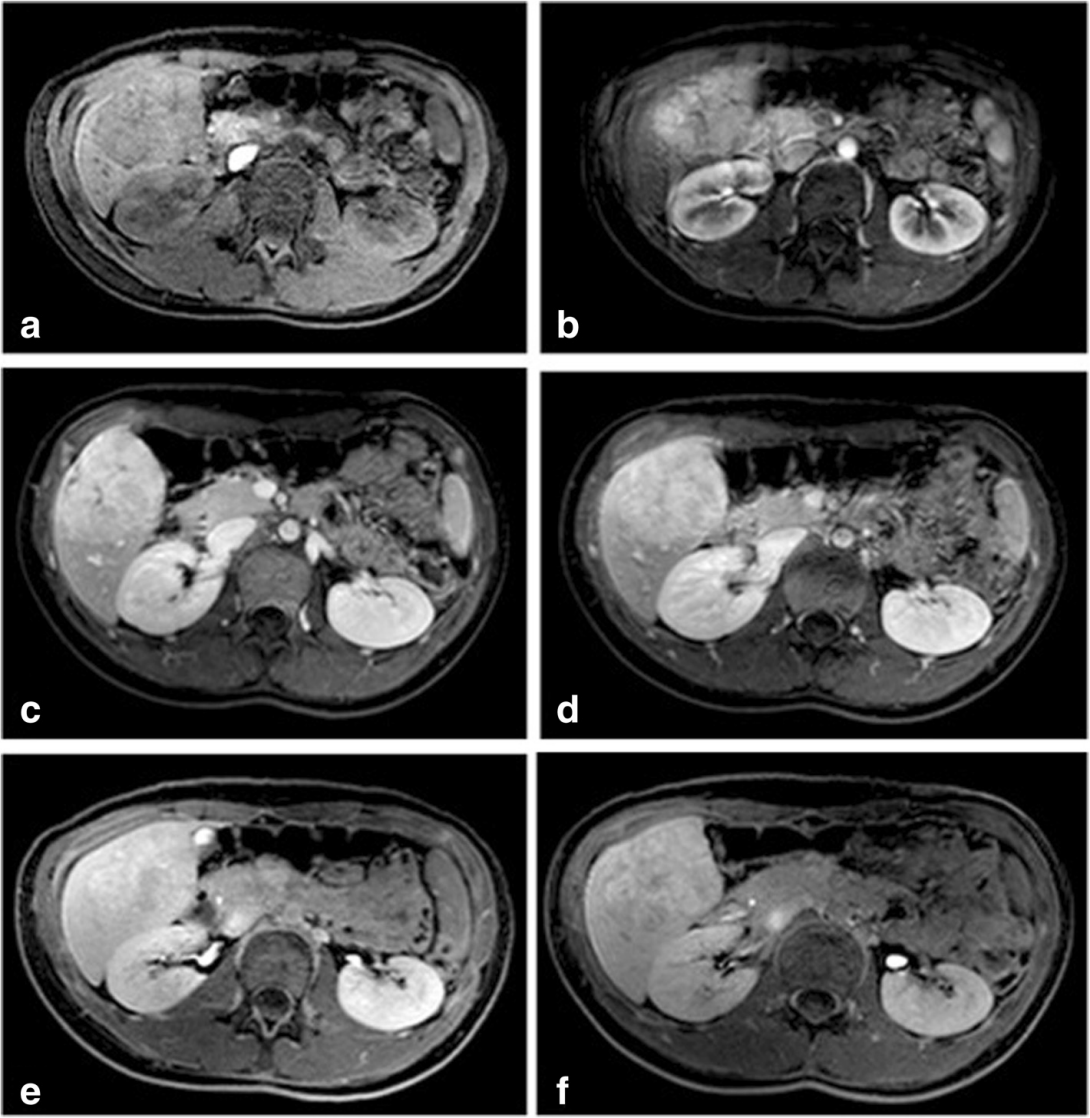
Focal nodular hyperplasia in a 14 year old female patient. The hepatic lesion is hypointense to background liver parenchyma on (**a**) pre-contrast T1-weighted fat-saturated sequences. There is avid enhancement of the lesion post gadobenate dimeglumine administration in (**b**) the arterial phase, with eventual homogenous enhancement of the lesion and central scar in the (**c**) portal venous and (**d**) equilibrium phases. The delayed (**e**) 30 min and (**f**) 45 min images show the lesion enhancing to a similar intensity as the background liver parenchyma

**Fig. 6:**
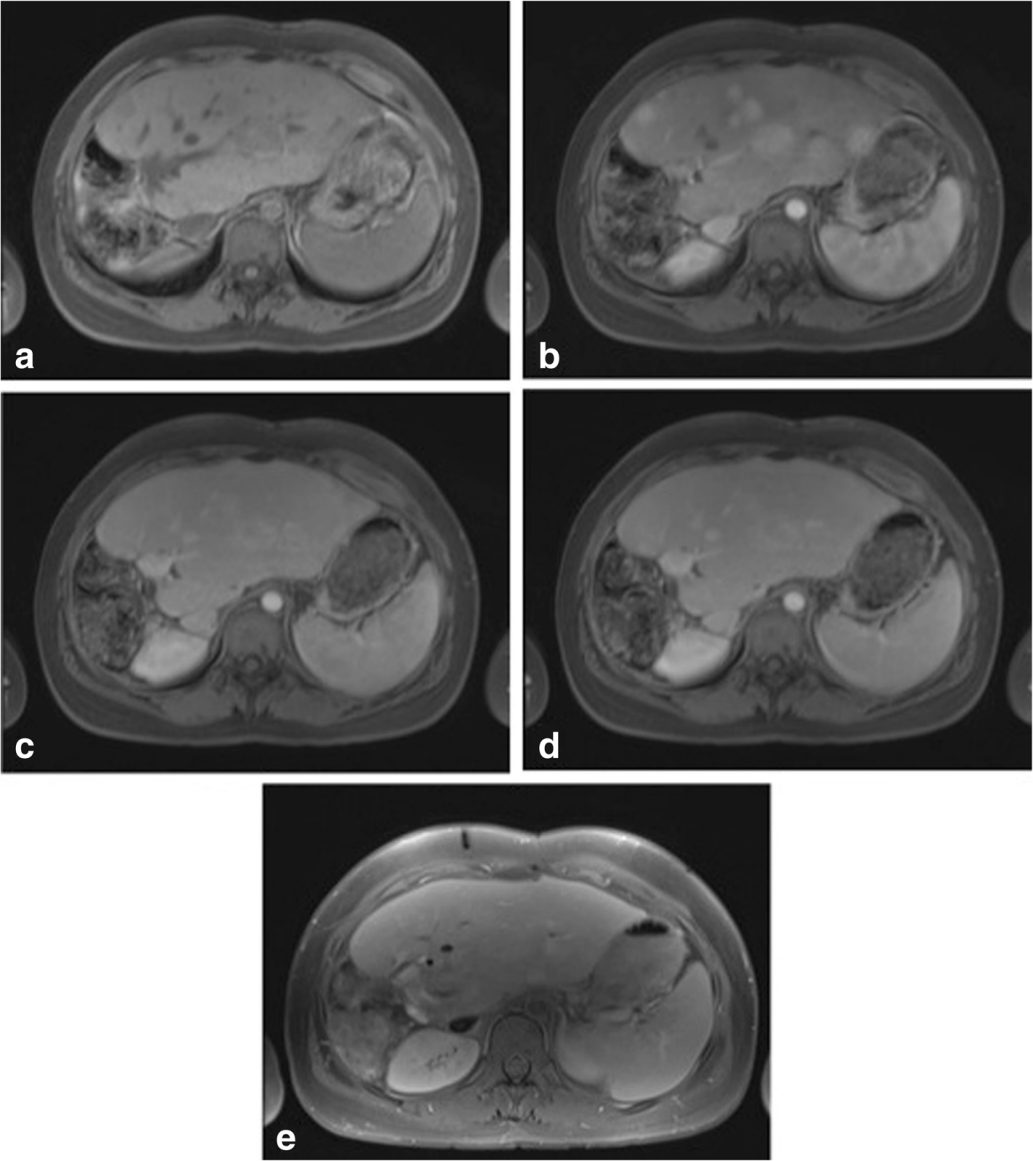
Multiple areas of focal nodular hyperplasia in a 17 year old patient with prior history of right hepatectomy for hepatoblastoma. Same patient as in Fig. 1. On the (**a**) pre-contrast T1-weighted fat-saturated images, the liver lesions are not visualized. Post gadobenate dimeglumine administration in (**b**) arterial phase imaging, there are multiple avidly enhancing lesions throughout the liver. These demonstrate similar internal signal intensity to the background liver parenchyma in (**c**) portal venous, (**d**) equilibrium and (**e**) 40 min delayed phases of imaging. There is no wash out of contrast material to suggest metastatic disease

As previously mentioned, mixed hepatocyte specific/extracellular agents are useful in helping to differentiate FNHs from other hepatic lesions. The presence of normal functioning hepatocytes within the FNH allow uptake of this contrast. There is enhancement of the FNH in the arterial phase due to contrast material leaking from the vascular space into the interstitial space, however during the hepatobiliary phase of imaging there is active hepatocellular uptake (depending on presence of transporter proetein OATP1B1/3 [47]) and therefore persistent lesion enhancement (Fig. 5). This feature helps to differentiate FNH from other malignant lesions, which typically demonstrate contrast wash-out when compared to normal liver parenchyma [20] (Fig. 6).

A pitfall in interpretation may occur when trying to differentiate FNHs from regenerative nodules, as both can be hyperintense in the hepatobiliary phase. Whilst pre-contrast features can sometimes be helpful in making the differentiation, it may be difficult in scenarios where there is haemorrhage or fatty deposition within the FNH, or in the presence of background liver disease. Lesions that do not conform to those of a typical FNH therefore warrant biopsy [18].

Hepatocellular adenomas also contain hepatocytes (like FNH), although not malformed biliary ducts. Fat content may be present within them, but is non-specific for the diagnosis [46]. Currently, four subtypes of hepatocellular adenomas are recognized: inflammatory, hepatocyte nuclear factor 1 alpha (HNF-1α) mutated, β-catenin mutated and unclassified. Each subtype has unique clinical, imaging and/or histopathologic findings [48, 49]. Adenomas have variable signal characteristics based on internal fat content, hemorrhage and histologic subtype. It should be noted that with the exception of the β-catenin (most commonly seen in the paediatric population) and inflammatory subtypes, most other hepatocellular adenoma subtypes are hypointense to the surrounding normal liver during the hepatocyte phase allowing radiologists to differentiate these lesions from FNH. Inflammatory and β-catenin subtypes of adenomas may appear iso/hyperintense during the hepatocyte phase and can be difficult to distinguish from FNH [50, 51].

## Conclusions

MR imaging for characterising paediatric liver tumours provides excellent soft tissue contrast. The usage of mixed hepatocyte specific/extracellular contrast agents allows for better lesion characterisation and location, particularly with respect to the biliary system and for differentiating FNH from other liver lesions.

Imaging should aim to clarify the presence of a lesion, the likelihood of malignancy and potential for complete surgical resection. Reviewing and reporting the studies should address these issues in a systematic fashion whilst also commenting upon background liver parenchymal appearances. Clinical information and adequate patient preparation prior to MR imaging studies help enhance the diagnostic yield.

## Abbreviations

ADC: Apparent Diffusion Coefficient
AFP: Alpha-Fetoprotein
CEUS: Contrast Enhanced Ultrasound
CT: Computed Tomography
FAP: Familial Adenomatous Polyposis
FNH: Focal Nodular Hyperplasia
GRE: Gradient Echo
HCC: Hepatocellular Carcinoma
HNF: Hepatocyte Nuclear Factor
IVC: Inferior Vena Cava
MIBG: Metaiodobenzylguanidine
MRI: Magnetic Resonance Imaging
PRETEXT: Pretreatment Extent of Tumour
SIOPEL: Société Internationale d'Oncologie Pédiatrique
SSFP: Steady State Free Precession
US: Ultrasound
